# Initial Severity of Injury Has Little Effect on the Temporal Profile of Long-Term Deficits in Locomotion, Anxiety, and Cognitive Function After Diffuse Traumatic Brain Injury

**DOI:** 10.1089/neur.2022.0057

**Published:** 2023-01-18

**Authors:** Frances Corrigan, Alina Arulsamy, Sandy R. Shultz, David K. Wright, Lyndsey E. Collins-Praino

**Affiliations:** ^1^ Head Injury Lab, Division of Health Sciences, University of South Australia, Adelaide, South Australia, Australia.; ^2^ Cognition, Ageing and Neurodegenerative Disease Lab, School of Biomedicine, The University of Adelaide, Adelaide, South Australia, Australia.; ^3^ Department of Neuroscience, Central Clinical School, Monash University, Melbourne, Victoria, Australia.; ^4^ Health and Human Services, Vancouver Island University, Nanaimo, British Columbia, Canada.

**Keywords:** anxiety-like behavior, cognition, locomotion, MRI, traumatic brain injury

## Abstract

Traumatic brain injury (TBI) is associated with persistent impairments in multiple domains, including cognitive and neuropsychiatric function. Previous literature has suggested that the risk of such impairments may differ as a function of the initial severity of injury, with moderate-severe TBI (msTBI) associated with more severe cognitive dysfunction and mild TBI (mTBI) associated with a higher risk of developing an anxiety disorder. Despite this, relatively few pre-clinical studies have investigated the time course of behavioral change after different severities of injury. The current study compared the temporal profile of functional deficits incorporating locomotion, cognition, and anxiety up to 12 months post-injury after an mTBI, repeated mild TBI (rmTBI), and single msTBI in an experimental model of diffuse TBI. Injury appeared to alter the effect of aging on locomotor activity, with both msTBI and rmTBI rats showing a decrease in locomotion at 12 months relative to their earlier performance on the task, an effect not observed in shams or after a single mTBI. Further, mTBI seemed to be associated with decreased anxiety over time, as measured by increased time spent in the open arm of the elevated plus maze from 3 to 12 months post-injury. No significant findings were observed on spatial memory or volumetric magnetic resonance imaging. Future studies will need to use a more comprehensive behavioral battery, capable of capturing subtle alterations in function, and longer time points, following rats into old age, in order to more fully assess the evolution of persistent behavioral deficits in key domains after different severities of TBI, as well as their accompanying neuroimaging changes. Given the prevalence and significance of such deficits post-TBI for a person's quality of life, as well as the elevated risk of neurodegenerative disease post-injury, such investigations may play a critical role in identifying optimal windows of therapeutic intervention post-injury.

## Introduction

Traumatic brain injury (TBI) is the result of a mechanical impact to the head, with severity ranging from mild to more severe injuries with prolonged loss of consciousness. Evidence suggests that the pattern of functional deficits and trajectory of recovery depends on severity of the initial injury and, in regard to mild TBI (mTBI), the number of impacts received.^1–3^ Single mTBI impairs numerous cognitive domains, including learning and memory, attention, and processing speed,^1^ with return to baseline functioning typically within 2–4 weeks,^4,5^ although a small percentage (∼15%) may show persistent cognitive symptoms.^6^ Repeated mTBIs (rmTBIs) within a short period of time appear to lengthen this recovery period.^4,5^ Moderate-severe TBI (msTBI) also impacts attention and speed of processing, psychomotor skills, learning and memory, verbal and visuospatial skills, and a range of executive functions.^7,8^ Recovery is less robust, with 50% of moderate TBI sufferers showing a degree of cognitive impairment at 12 months post-injury.^9^ This is significant, given that persistent impairments in cognition are a major predictor of quality of life in persons after a TBI.^10,11^

Interestingly, in contrast to cognition, where cognitive impairments are enhanced with increased severity of injury, post-TBI anxiety disorders appear to be more prevalent post-mTBI.^12^ Conversely, greater disability at 1 year after a moderate-severe TBI was actually associated with lower scores on both the Anxiety and Anxiety-related Disorders subscales of the Personality Assessment Inventory, although this may be attributable to poor insight into psychological functioning in these persons.^13^ Nonetheless, TBI, regardless of severity, is linked to a lifelong increased risk of experiencing clinically significant anxiety,^14^ with prevalence rates of up to 70% reported.^15^ This is significant, given that such disorders can be difficult to treat and may require long-term management.^16^

Given the evidence that initial injury severity can alter behavioral outcomes long term post-TBI, it is critical to understand the brain mechanisms that may drive this, for which pre-clinical studies are needed. Despite this, many pre-clinical studies to date fail to assess behavioral changes >12 weeks post-injury. Thus, this study compared the temporal profile of functional deficits incorporating locomotion, cognition, and anxiety up to 12 months post-injury after a single mild, repeated mild, and single moderate-severe injury in a model of experimental diffuse TBI. Further, to investigate whether there were any changes in volume in key brain regions known to underlie these behaviors, brains were collected at 12 months post-injury and *ex vivo* magnetic resonance imaging (MRI) was performed.

## Methods

To investigate, male Sprague-Dawley rats (10–12 weeks; 400–450 g) were used under approval of the University of Adelaide Animal Ethics Committee (M-2015-243A and M-2015-187). Rats were housed under conventional laboratory conditions, with a 12-h light-dark cycle and access to food and water *ad libitum*. Rats were randomly allocated to receive either sham surgery (*n* = 7), repetitive sham surgery (three incisions at 5-day intervals; *n* = 7), or a single mTBI (*n* = 14), rmTBI (three mild diffuse injuries at 5-day intervals; *n* = 14), or msTBI (*n* = 14). This cohort of animals have previously had part of the 12-month data battery reported.^17^ Injury was induced using the Marmarou weight-drop model as previously described,^17^ with the 450-g weight dropped from 2 m for msTBI and 0.75 m for mTBI. Rats in the moderate/severe diffuse TBI group were also subjected to hypoxic conditions (2 L/min nitrogen; 0.2 L/min oxygen) for 10 min post-injury, to replicate the clinical effects observed following this injury model without ventilation, given that this hypoxic condition is known to exacerbate injury severity.^18^

Functional tests assessing cognition (spatial working memory as measured by the Y-maze),^19^ anxiety (as measured by both the elevated plus maze [EPM] and time in centre in the open field test [OFT]),^20^ and locomotion (as measured by distance traveled in the OFT)^21^ were performed at 7 days and at 1, 3, 6, and 12 months post-injury within the same animal cohort. All functional data were recorded using the ANY-maze Video Tracking System (version 4.99m; Stoelting Co., Wood Dale, IL). For the OFT, rats were placed in the center of a large square box (95 × 95 cm^2^), with walls at a height of 44.5 cm, and the total distance traveled over a 5-min period was recorded. Time in the center of the field was also measured for anxiety-like behavior. For the EPM, rats were placed in the center of an elevated (50 cm in height) cross-shaped maze consisting of two open and two closed (walls of height, 40 cm) maze arms (each of a length of 50 cm), facing the open arms, for 5 min. Time spent in the open arms measured by the center point of the animal's body was recorded, with increased time spent in the closed arms thought to represent anxiety-like behavior.

For the Y-maze, rats were placed in an equally angled Y-shaped arena, with each arm of the maze identical in size and shape, but visually distinct (because of cues on the wall). In the first exposure, one arm was closed off. One hour later, rats were reintroduced to the maze with all arms now open, and time spent exploring each arm was recorded. In each phase, animals were placed in the maze for 3 min. However, given that exploration was markedly decreased after the first minute, the percentage of animals in each group entering the novel arm first was also calculated, as was the latency to enter the novel arm.

Rats were perfusion-fixed with 10% formalin, and brains were removed and prepared for *ex vivo* imaging as described previously.^22^ A subset of animals underwent *ex vivo* imaging (sham = 6, rmTBI = 7, mTBI = 6, and msTBI = 8). MRI was performed with a 9.4/20 Bruker instrument (Bruker, Billerica, MA) and actively decoupled volume transmit and phased-array surface receive coils. A three-dimensional T2*-weighted image was acquired in the axial plane with a multiple-gradient-echo sequence with the following parameters: repetition time = 68 ms; first echo time = 2.7 ms; echo-spacing = 3.75 ms; number of echoes = 14; field of view = 30.72 × 19.52 × 12.8 mm^3^; matrix size = 192 × 122 × 80; and isotropic spatial resolution = 160 × 160 × 160 μm^3^. Images were reconstructed using an in-house MATLAB (version R2021a; The MathWorks, Natick, MA) code and the mean echo time image registered to the Waxholm Space (WHS) atlas of the Sprague-Dawley rat brain, downsampled to 78 × 78 × 78 μm.^323^ Registration was performed using symmetric normalization with cross-correlation^24^ and the resulting diffeomorphisms used to register the WHS labels to subject space.

Volume was calculated for the WHS-defined dentate gyrus, cornu ammonis (as a combination of regions 1, 2, and 3), amygdala, infralimbic, pre-limbic, and M1 area labels using MATLAB. These brain regions were selected for analysis because they are known to be critical for driving the behaviors assessed. Specifically, several brain regions, including the hippocampus and pre-frontal cortex, are known to underlie performance on the Y-maze spatial working memory task,^25^ leading to selection of the dentate gyrus, cornu ammonis, and infra- and pre-limbic areas for analysis. These regions have also been shown to be implicated in performance on both the EPM and OFT.^26,27^ Similarly, the amygdala is known to play a key role in anxiety-related behaviors in rodents, with distinct subregions of the amygdala differentially modulating behavior in these tests (for review, see La-Vu and colleagues).^28^ Finally, given its role in the execution of voluntary motor function and regulation of locomotor activity, the primary motor cortex (M1) was assessed.^29^

All behavioral data were analyzed by repeated-measures two-way analysis of variance (ANOVA), and MRI volumetric measures were assessed by one-way multiple ANOVA using IBM SPSS statistics (version 24; IBM Corp., Armonk, NY) and GraphPad Prism software (GraphPad Software Inc., La Jolla, CA). *p* values <0.05 were considered statistically significant. Shams and repetitive shams were combined together as a single sham group, given that they did not differ significantly in any parameters measured (*p* > 0.05).

## Results

**FIG. 1.**
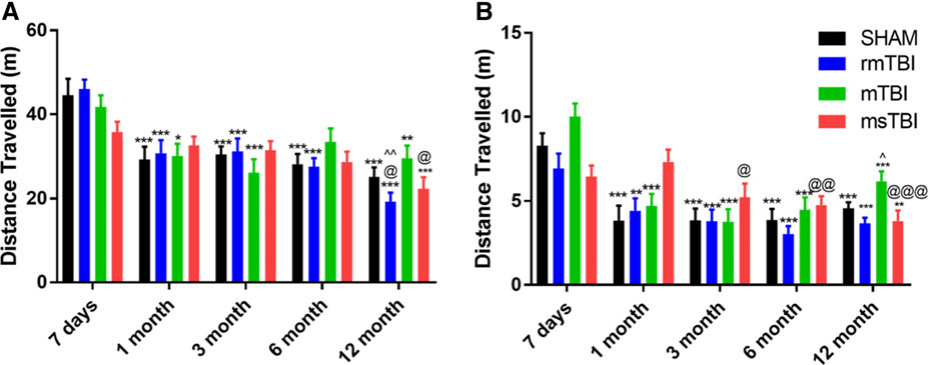
Distance traveled (m) as a measure of locomotion on the (**A**) open field maze and (**B**) elevated plus maze. Graphs represent the mean ± SEM (***p < 0.001, **p < 0.01 vs. 7-day time point; ^@^*p* < 0.05, ^@@^*p* < 0.01, ^@@@^*p* < 0.001 vs. 1-month time point; ^^^*p* < 0.05, ^^^^*p* < 0.01 compared to 3-month time point; *n* = 12–14 per group). mTBI, mild traumatic brain injury; msTBI, moderate-severe traumatic brain injury; SEM, standard error of the mean.

General locomotor activity was assessed as distance traveled in the OFT (Fig. 1A). There was a significant effect of time post-injury on locomotion (*F*_4,200_ = 24.85, *p* < 0.0001). Although there was no significant injury severity effect (*F*_3,50_ = 0.345, *p* = 0.793), there was a significant interaction effect (*F*_12,200_ = 2.098, *p* = 0.02). Sham rats had a significant decrease in locomotion from 7 days to 1 month post-injury (41.10 ± 4.56 vs. 26.98 ± 3.46 m; *p* < 0.001) and then maintained similar locomotion out to 12 months (26.09 ± 1.97 m), such that the 1-, 3-, 6-, and 12-month time points were all significantly decreased compared to 7 days (*p* < 0.001). mTBI rats showed a similar pattern, with significant differences between 1-, 3-, and 12-month rats compared to 7 days (*p* < 0.05).

In rmTBI rats, a decrease in locomotion was also noted from 7 days to 1 month post-injury (46.04 ± 2.26 vs. 30.74 ± 3.07 m; *p* < 0.001), which was sustained out to 6 months (27.49 ± 2.02), with a further decrease in locomotion at 12 months post-injury (19.22 ± 2.18 m), such that significant differences were observed between the 1-, 3-, 6-, and 12-month time points compared to 7 days (*p* < 0.001) and the 1- and 3-month compared to the 12-month time points (*p* < 0.05). In comparison, msTBI rats did not show a decrease in locomotion from 7 days to 1 month post-injury (35.75 ± 2.48 vs 32.55 ± 7.93 m; *p* = 0.88) and, instead, displayed a gradual decline in locomotion, such that only the 12-month rats traveled significantly less than the 7-day and 1-month cohorts (22.30 ± 2.60 m; *p* < 0.05).

This pattern (Fig. 1B) was similarly reflected by distance traveled in the EPM (time post-injury: *F*_4,188_ = 42.99, *p* < 0.001; severity of injury: *F*_4,188_ = 1.49, *p* = 0.23; interaction effect: *F*_12,188_ = 5.42, *p* < 0.001). Sham, rmTBI, and mTBI rats all decreased in locomotion after 7 days, such that they traveled significantly less at 1, 3, 6, and 12 months post-injury than at 7 days (*p* < 0.05). In contrast, msTBI rats had similar levels of exploration at 7 days and 1 month post-injury, with a decrease noted thereafter, such that distance traveled was less at 3, 6, and 12 months than at 1 month post-injury (*p* < 0.05).

**FIG. 2.**
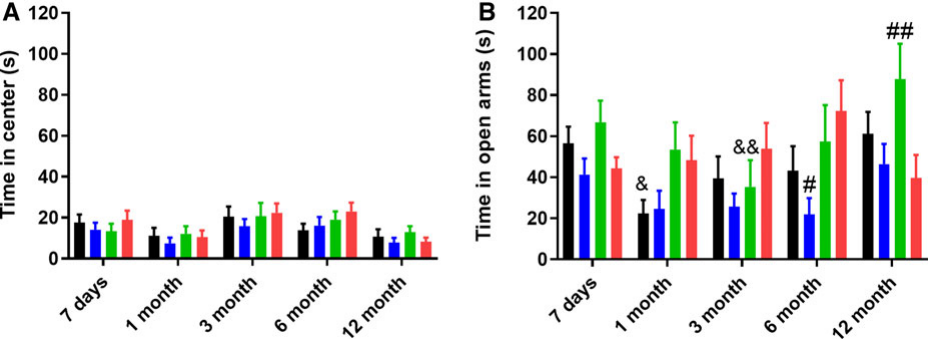
Anxiety-like phenotype as measured by time spent (s) in the (**A**) center of the open field and (**B**) open arms of the elevated plus maze. Graphs represent the mean ± SEM (^&^*p* < 0.05, ^&&^*p* < 0.01 vs. 12-month time point; ^#^*p* < 0.05, ^##^*p* < 0.01 compared to the msTBI group at that time point; *n* = 12–14 per group). msTBI, moderate-severe traumatic brain injury; SEM, standard error of the mean.

Anxiety-like behavior was measured by time spent in the center of the OFT (Fig. 2A), which showed a significant effect of time post-injury (*F*_4,200_ = 7.23, *p* < 0.0001), although neither injury effect (*F*_3,50_ = 0.59, *p* = 0.62) nor interaction (*F*_12,200_ = 0.52, *p* = 0.90) were significant. In contrast, when examining anxiety by time in the open arms of the EPM (Fig. 2B), a significant main effect of both time post-injury (*F*_4,188_ = 3.5, *p* = 0.008) and injury severity (*F*_4,188_ = 3.03, *p* = 0.04), as well as a significant interaction between the two (*F*_12,188_ = 1.90, *p* = 0.04) were noted. Sham rats showed a trend toward significance in decreased time in the open arms from 7 days to 1 month (63.26 ± 29.10 vs. 24.13 ± 26.03; *p* = 0.07), with a gradual increase in the open arms over the rest of the testing period, such that more time was spent in the open arms at 12 months compared to 1 month (24.13 ± 26.03 vs. 66.40 ± 40.46; *p* < 0.05).

In contrast, mTBI rats did not show a substantial decrease in time spent in the open arms at 1 month post-injury (67.29 ± 33.27 vs. 50.38 ± 43.10). Time in the open arms further increased at 12 months post-injury, such that mTBI rats spent significantly more time in the open arms than at 3 months post-injury (81.80 ± 58.28 vs. 36.74 ± 41.40; *p* < 0.05). At this 12-month time point, mTBI rats also spent significantly more time than msTBI rats in the open arms (81.80 ± 58.28 vs. 39.68 ± 40.16; *p* < 0.05). No significant fluctuations in performance over the 12-month period were observed in either the msTBI or rmTBI groups, although, at 6 months post-injury, rmTBI rats spent significantly less time in the open arms than msTBI rats (72.24 ± 54.18 vs. 15.59 ± 17.60).

**FIG. 3.**
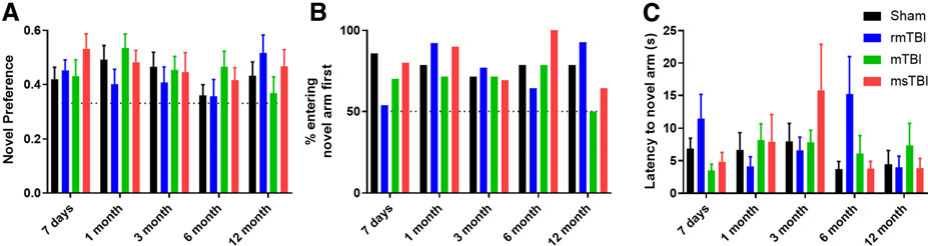
Cognition assessed through the Y-maze for spatial working memory measured by novel preference (**A**), percentage of animals entering the novel arm first (**B**), and latency to enter the novel arm (**C**). Graphs represent the mean ± SEM; *n* = 12–14 per group. mTBI, mild traumatic brain injury; msTBI, moderate-severe traumatic brain injury; SEM, standard error of the mean.

Cognitive outcome was assessed using the Y-maze for spatial memory (Fig. 3). No effect of either injury (*F*_3,50_ = 0.49; *p* = 0.69) or time (*F*_4,200_ = 1.6, *p* = 0.17) was noted in novel preference in the first minute within the maze (Fig. 3A). Across the time points assessed, 71–85% of the sham animal group chose to enter the novel arm first, with similar performance observed in the other groups (Fig. 3B). Indeed, no difference in latency to enter the novel arm was noted, with no effect of injury (*F*_3,50_ = 0.36, *p* = 0.79) or time (*F*_4,200_ = 1.26, *p* = 0.29; Fig. 3C).

**FIG. 4.**
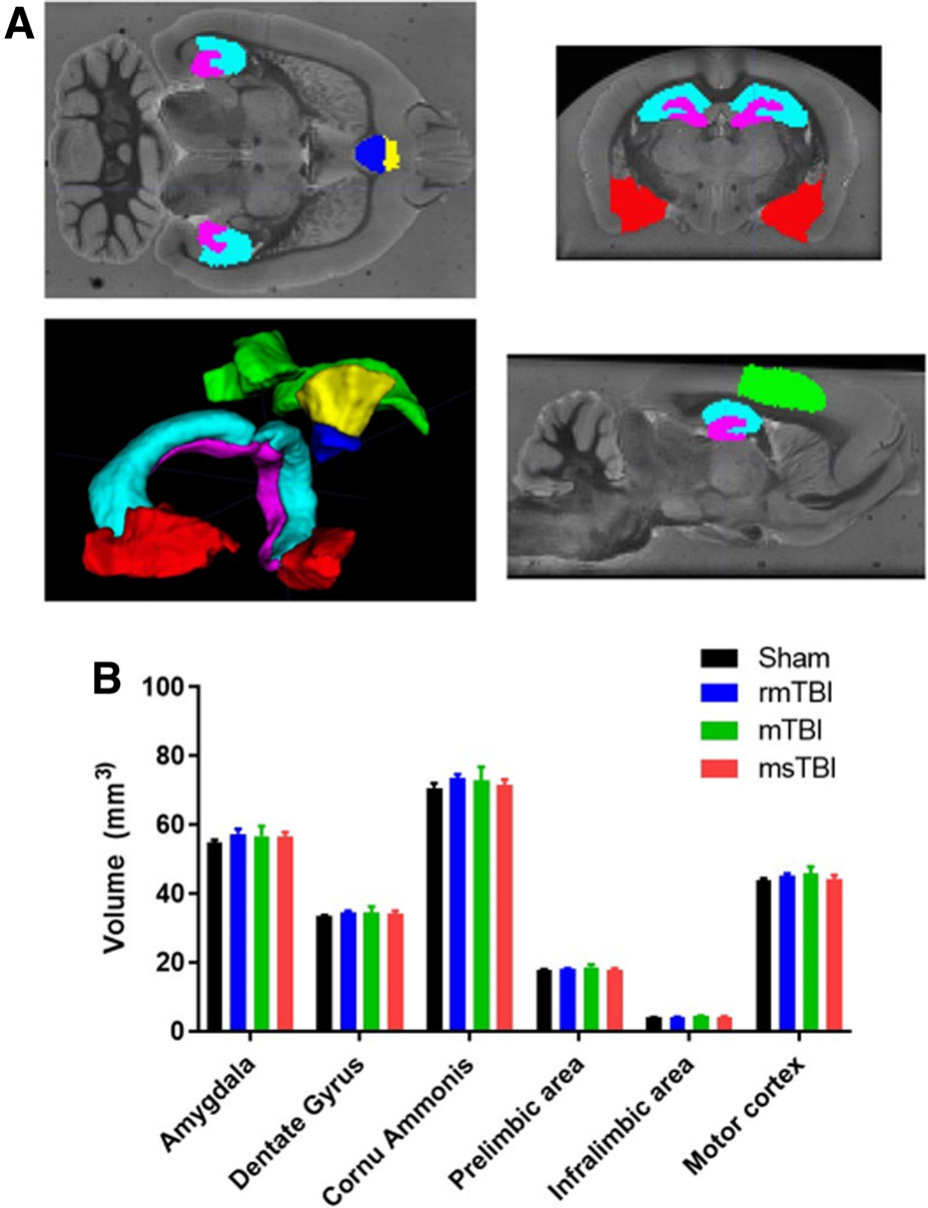
(**A**) Example image showing the WHS-defined regions registered to an example subject's bias field-corrected mean echo image, with volumetric rendering of the regions. Red = amygdala; magenta = dentate gyrus; cyan = cornu ammonis; yellow = pre-limbic area; blue = infralimbic area; and green = M1. (**B**) Analysis of volumetric data in the regions of interest showing no difference between injury groups at the 12-month time point. Graph represents the mean ± SEM (*n* = 6–8 per group). mTBI, mild traumatic brain injury; msTBI, moderate-severe traumatic brain injury; SEM, standard error of the mean; WHS, Waxholm Space.

No changes in brain volume were noted in any structure examined at 12 months post-injury, regardless of injury severity. This included the primary motor cortex (*F*_3,23_ = 0.624, *p* = 0.61), hippocampus/dentate gyrus (*F*_3,23_ = 0.364, *p* = 0.78) and subfields (*F*_3,23_ = 0.40, *p* = 0.75), pre-limbic (*F*_3,23_ = 0.679, *p* = 0.57) and infralimbic (*F*_3,23_ = 0.721, *p* = 0.55) areas, or the amygdala (*F*_3,23_ = 0.378, *p* = 0.77) (Fig. 4).

## Discussion

The current study investigated the presence of functional impairments and gray matter volume changes from 7 days to 12 months post-TBI of different severities: mTBI, rmTBI, and msTBI. Surprisingly, no injury effect relative to shams was noted on anxiety, as measured by both time in center of the open field and time spent in the open arms of the EPM, nor in locomotor activity within the OFT and EPM. Injury did appear to alter the trajectory of aging on locomotion, with msTBI and rmTBI rats showing a decrease in locomotion at 12 months relative to their earlier performance on the task, an effect not observed in shams or after a single mTBI. Further, there appeared to be an effect of mTBI on decreasing anxiety, with increased time in the open arms of the EPM from 3 to 12 months post-injury. Volumetrics analysis with *ex vivo* MRI revealed no difference in volume of key gray matter structures at 12 months post-injury.

Few studies have utilized the Y-maze to examine long-term spatial recognition memory post-TBI, with deficits noted up to 2 weeks after a single focal controlled cortical impact injury^30^ and up to 3 months post-rmTBI utilizing this diffuse injury model in our own laboratory.^31^ In this study, sham animals entered the novel arm first at a higher than chance rate, with latencies to enter the novel arm averaging <10 sec. However, the preference itself was modest, between 0.4 and 0.5, just higher than a 0.33 baseline preference. Nonetheless no difference in this performance was noted in any of the injury groups, nor was there an effect of age. Indeed, our studies are in contrast to previous pre-clinical studies suggesting that repeated diffuse impacts may lead to hippocampal-driven spatial memory deficits, with impairment on the Morris water maze noted up to 6 months post-injury after three impacts from the CHIMERA model.^32^ Interestingly, msTBI may be associated with a different pattern of change. Spatial reference memory, and hence hippocampal function, are intact up to 12 months after diffuse axonal injury.^17^

This is consistent with previous studies of diffuse moderate-severe TBI, which have shown a lack of cognitive deficits on either the Morris water maze or radial arm maze,^33,34^ with a concomitant lack of hippocampal cell loss^33^ and preservation of hippocampal synaptic proteins,^35^ after impact-acceleration TBI. Future studies incorporating a wider battery of functional tests, with more sensitive measures of cognitive function, are required to more fully assess this. Alternatively, the time frame utilized, which equated roughly to later middle age in humans, may have been insufficient, with recent work finding that it is not until 24 months post-injury that spatial memory deficits emerge after both a single and repeated mild TBI.^36^

Similar to our cognitive results, no significant differences relative to sham rats were noted in anxiety-like behavior in terms of either time spent in the center of the open field or time in the open arms of the EPM. However, mTBI rats spent more time in the open arms at 12 months than msTBI rats, with significantly increased exploration from 3 to 12 months post-injury, suggestive of decreased anxiety. This would be in line with previous reports that mTBI, in particular, is associated with decreased anxiety on the EPM,^37,38^ with this attributed to increased risk-taking behavior and/or increased impulsivity. Interestingly, other studies have also reported this behavior after an rmTBI, with increased exploration of the open arm noted up to 8 months post-injury,^39–41^ an effect we did not observe here.

Indeed, rmTBI rats actually spent significantly less time in the open arm than msTBI rats at 6 months post-injury. This may reflect differences in injury schedules (number of injuries, timing between insults, and location injured), with further research needed to investigate this. In contrast, msTBI alone had minimal effects on anxiety, with no differences relative to sham rats. This is in line with other reports subsequent to a similar type of injury at 7 weeks post-injury,^42^ although mixed injury models, such as fluid percussion, report increased anxiety to 6 months post-injury, suggesting that this may be dependent on the nature of the injury.^43^

It should be noted that a significant interaction effect was noted in distance traveled in both the OFT and EPM, with msTBI rats showing a different pattern over time in locomotor activity post-injury. All other groups had a decrease in locomotor activity from the 7-day to 1-month time points, with persistently reduced locomotion after the first exposure. In contrast, in the open field, msTBI rats had a similar performance from 7 days to 6 months post-injury, with a decrease in locomotion only noted at the 12-month time point. Similarly, in the EPM, distance traveled only decreased from the 3-month time point, with comparable levels of activity observed from 7 days to 1 month post-injury. The decrease in locomotor activity noted within the sham, rmTBI, and mTBI groups most likely reflects habituation, where familiarity with the task reduces its novelty and hence the drive to explore.^44,45^ Given that msTBI rats did not show this habituation effect after the first exposure, this could suggest impaired spatial memory.^46,47^ Alternatively, given the greater motor impairment noted immediately after an msTBI as seen as a reduction in rotarod scores,^17^ this may have reduced their locomotor ability at 7 days, precluding the ability to explore sufficiently in order to observe a habituation effect at later time points.

The other notable effect observed in locomotor activity within the open field was that both the rmTBI and msTBI groups showed a decrease in locomotor performance at the 12-month time point relative to their earlier activity levels, an effect not observed in the sham or mTBI rats. This may reflect an acceleration of the effects of aging on locomotion subsequent to this type of injury, with age known to decrease locomotor activity of rodents in the open field,^48^ analogous to decreased locomotor activity observed clinically with age.^49,50^ Previous clinical imaging studies have suggested that moderate-severe, but not mild, TBI accelerates aging, with a predictive model of aging using machine learning of MRI images finding that those with a history of msTBI had an estimated brain age of ∼5 years greater than their chronological age.^51^ Nonetheless, given the lack of significance noted relative to shams, or the failure to observe any other effects in the other domains examined here, future studies incorporating later time points would be required to confirm this hypothesis.

The lack of overt behavioral differences noted to 12 months post-injury was supported by volumetric analysis indicating no structural loss at this time point. Interestingly, at 30 days post-msTBI with the same weight-drop model, reduced volume in both the subiculum and posterior dorsal hippocampus was noted,^52^ indicating that subregional analysis may be required to find subtle alterations. However, in this study, we separated the hippocampus into separate regions for the dentate gyrus and cornu ammonis, but still did not observe an effect of injury at the 12-month time point. Mixed focal/diffuse models typically show more extensive changes, with, for example, the lateral fluid percussion model showing progressive volume reduction in both gray and white matter regions, such as the hippocampus, cortex, and corpus callosum, up to 1 month post-injury.^53,54^ Instead, it is possible that diffuse axonal injury may be associated with more subtle underlying changes. In line with this, diffuse axonal injury can be challenging to identify using traditional methods, such as conventional computed tomography or MRI.^55^ Given this, more novel imaging techniques, including susceptibility-weighted imaging and diffusion tensor imaging (DTI), may be needed to identify the changes that occur long term subsequent to diffuse TBI and investigate how these correlate with changes in behavior.^56^ Nevertheless, the lack of overt gray matter volume differences within key brain regions at 12 months post-injury observed in this study provides further support that there is no evidence of neurodegeneration present at this late-middle-age time point. with further aging perhaps needed to observe such a change.

This was the first study to track performance on behavioral tasks encompassing locomotion, anxiety, and spatial memory in the same cohort of rats up to 12 months post-injury and investigate whether this was associated with underlying changes in gray matter volume within related brain regions. In line with our previous study at the 12-month time point,^17^ we observed no direct effects (relative to shams) of any of the injury types on any of the parameters measured. Further, we did not observe any gray matter volume loss at this time point. This suggests that any acute effects of injury had resolved by 7 days and that the time frame utilized was not sufficient to note the re-emergence of any deficits nor accompanying volumetric change.

Alternatively, other behavioral domains not measured here, such as depression or executive function, may be more susceptible to diffuse TBI, whether single mild, single moderate-severe, or repeated impacts, and thus a more extensive and sensitive behavioral testing battery may be necessary to detect the subtle cognitive deficits likely to be present at these time points. Similarly, alternative imaging techniques, such as white matter analysis using DTI, may be necessary in order to detect subtle changes in underlying neuronal circuitry. Given the prevalence and significance of persistent deficits post-TBI for a person's quality of life, as well as the known elevated risk for neurodegenerative disease development post-injury,^57^ understanding how deficits in key domains affected by TBI evolve over time subsequent to different severities of injury may be a key step toward identifying optimal windows of therapeutic intervention, and thus future work in this area is critical.

## Abbreviations Used

ANOVA: analysis of variance
DTI: diffusion tensor imaging
EPM: elevated plus maze
MRI: magnetic resonance imaging
msTBI: moderate-severe TBI
mTBI: mild TBI
OFT: open field test
rmTBI: repeated mild TBI
TBI: traumatic brain injury
WHS: Waxholm Space
